# Pulpbow: A Method to Study the Vasculogenic Potential of Mesenchymal Stem Cells from the Dental Pulp

**DOI:** 10.3390/cells10112804

**Published:** 2021-10-20

**Authors:** Andrea Mantesso, Zhaocheng Zhang, Kristy A. Warner, Alexandra E. Herzog, Ajai J. Pulianmackal, Jacques E. Nör

**Affiliations:** 1 Angiogenesis Research Laboratory, Department of Cariology, Restorative Sciences and Endodontics, University of Michigan School of Dentistry, Ann Arbor, MI 48109, USA; mantesso@umich.edu (A.M.); zczhang@umich.edu (Z.Z.); kawarner@umich.edu (K.A.W.); aoklejas@umich.edu (A.E.H.); 2 Department of Molecular, Cellular and Developmental Biology, University of Michigan College of Literature, Science and the Arts, Ann Arbor, MI 48109, USA; ajai@umich.edu; 3 Department of Biomedical Engineering, University of Michigan College of Engineering, Ann Arbor, MI 48109, USA; 4 Department of Otolaryngology, University of Michigan School of Medicine, Ann Arbor, MI 48109, USA

**Keywords:** vasculogenesis, angiogenesis, tissue regeneration, endothelial cells, cell fate, stemness, differentiation

## Abstract

Understanding how Mesenchymal Stem Cells (MSCs) form blood vessels is critical for creating mechanism-based approaches for the therapeutic use of these cells. In addition, understanding the determinants and factors involved in lineage hierarchy is fundamental to creating accurate and reliable techniques for the study of stem cells in tissue engineering and repair. Dental Pulp Stem Cells (DPSC) from permanent teeth and Stem cells from Human Exfoliated Deciduous teeth (SHED) are particularly interesting sources for tissue engineering as they are easily accessible and expandable. Previously, we have shown that DPSCs and SHEDs can differentiate into endothelial cells and form functional blood vessels through vasculogenesis. Here, we described how we created the “pulpbow” (pulp + rainbow), a multicolor tag experimental model that is stable, permanent, unique to each cell and passed through generations. We used the pulpbow to understand how dental pulp stem cells contributed to blood vessel formation in 3D models in in vitro and ex vivo live cell tracking, and in vivo transplantation assays. Simultaneous tracking of cells during sprout formation revealed that no single multicolor-tagged cell was more prone to vasculogenesis. During this process, there was intense cell motility with minimal proliferation in early time points. In later stages, when the availability of undifferentiated cells around the forming sprout decreased, there was local clonal proliferation mediated by proximity. These results unveiled that the vasculogenesis process mediated by dental pulp stem cells is dynamic and proximity to the sprouting area is critical for cell fate decisions.

## 1. Introduction

Postnatal mesenchymal stem cell populations have been found in most healthy and non-healthy oral tissues. Among the most studied dental stem cell populations are the ones derived from the dental pulp of both permanent (DPSCs) and deciduous teeth (SHEDs). These populations are easily accessible and can be effortlessly isolated from routinely extracted premolars and 3rd molars or from exfoliating deciduous teeth [1,2]. Both SHEDs and/or DPSCs can be used to treat dental and non-dental conditions in preclinical studies [3,4,5] and to have higher proliferative rates [1] and higher regenerative capacity [6] than mesenchymal stem cells (MSCs) derived from the bone marrow. These characteristics facilitate their ex vivo expansion in sufficient numbers [7,8] making them excellent candidates for cell therapies and tissue engineering. Interestingly, the dental pulp and bone marrow are both highly vascularized innervated soft tissues, encircled by cells capable of forming mineral tissue [9]. In a living organism, cells reside within 100–200 µm of a capillary as they cannot survive outside of this range simply by diffusion [10]. In tissue engineering, the absence of a vascular network capable of distributing nutrients and oxygen is a major limiting factor [11]. In this regard, dental pulp stem cells (SHEDs and DPSCs) can specifically differentiate into vasculogenic endothelial cells both in vitro and in vivo [12,13,14], anastomize with the host vasculature [15] and have higher angiogenic activity than bone marrow MSCs [5]. Nevertheless, the precise cellular and molecular mechanisms by which dental pulp stem cells form functional blood vessels remain mostly unclear.

We previously have shown that VEGF (Vascular Endothelial Growth Factor) induces vasculogenic differentiation of dental pulp stem cells (DPSCs and SHEDs) via activation of the canonical Wnt/b-catenin signaling pathway [14]. Wnt alone can induce this differentiation while silencing Wnt blocks this process. Thus, dental pulp stem cells can be induced to differentiate into blood vessels in a process that resembles the embryonic vasculogenesis [14]. VEGF signals through VEGFR1 to activate MEK1/ERK signaling and inhibit STAT3 transcriptional activity resulting in endothelial differentiation of pulp stem cells [13]. A crosstalk between endothelial cells and stem cells within the perivascular niche is required for the maintenance of stem cell pools in dental pulps. Confocal microscopy showed that ALDH1^high^ and Bmi-1^high^ stem cells are preferentially localized in close proximity to blood vessels in physiological human dental pulps. In contrast, transplantation of dental pulp stem cells stably transduced with short hairpin RNA (shRNA)-STAT3 into immunodeficient mice revealed a decrease in the number of blood vessels surrounded by ALDH1^high^ or Bmi-1^high^ cells (perivascular niches) compared to tissues formed by control stem cells [16]. More recently, we showed that after a brief induction with VEGF, DPSCs start to express VEGFR2 [15].

Dental pulp stem cells are composed of different sub-populations and contain at least one unique subset of stem cells characterized by high VEGFR1 expression that are primed for vasculogenic differentiation. This sub-population of cells corresponds to 10–20% of DPSC cells that can differentiate into endothelial cells [17]. In addition, p53/p21 functioning through Bmi-1 can prevent the vasculogenic differentiation of DPSC cells. Thus, the regulation of the stemness state is also an important factor that can influence the capacity to undergo vasculogenic differentiation besides the percentage of stem cells that are “primed” to differentiate into vascular endothelial cells [18].

Lineage tracing is the identification of all progenies of a single cell and a powerful tool to understand complex biological processes that involve different cell hierarchies and multiple cell types [19], such as vasculogenesis. One of the most elegant lineage tracing strategies is the brainbow which uses the Cre-lox recombination system to insert multiple copies of a transgenic construct carrying three different main hues (RGB—red, blue and green) into the genome of a mouse. As a result, mouse brain cells express randomly different ratios of fluorescent proteins and exhibit a variety of colorful hues known as the rainbow effect [20,21]. Brainbow-like strategies are now used to create similar colorful cellular identification tags [22] in cultured cells. Instead of using Cre-recombination techniques, cells in culture can be transduced with lentiviral vectors (LeGo vectors—Lentiviral Gene Ontology Vectors) that encode RGB fluorescent proteins. The random combination of these three basic colors into the cells’ genome will also result in the whole spectrum of rainbow colors [22]. Herein we used the LeGo vectors to create the pulpbow and used this system to track dental pulp stem cell fate while they undergo vasculogenic differentiation and form blood vessels. We aimed to analyze how stem cells from permanent and deciduous pulp tissues contributed to tissue vascularization using 3D models in in vitro, ex vivo and in vivo assays.

## 2. Materials and Methods

### 2.1. Pulpbow

Prior to the creation of the pulpbow, DPSCs and SHED were cultured in growth medium composed of α-minimum essential medium (α-MEM; Invitrogen; Waltham, MA, USA) supplemented with 20% fetal bovine serum (FBS; Invitrogen), 1% antibiotic-antimycotic (Invitrogen) and 20 µg/mL Plasmocin (InVivogen; San Diego, CA, USA). Cells were passed according to the cellular metabolism and before reaching 80% confluency. SHED cells were developed and characterized according to the protocol previously published by the Songtao Shi laboratory [2].

To create the pulpbow, DPSCs I and II (Lonza; Basel, Switzerland) and SHED (gift from Songtao Shi) were transduced with LeGO vectors (Lentiviral Gene Ontology), as described by Weber and colleagues [22]. The plasmids used were commercially available lentiviral vectors constructed under the SFFV promoter (Addgene; Watertown, MA, USA): LeGO-Cer 2 (Cerulean34, blue, cat# 27338), LeGO-C2 (mCherry35, red, cat# 27339) and LeGO-V2 (Venus36, green, cat# 27340). For the transfection, a cocktail composed of 2 M CaCl_2_, 2× HBS (Hepes Buffered Saline), double distilled water and a DNA mixture of 10 ug of psPAX2, 5 ug of pMD2.G and 3 µg of each 3 LeGo lentiviral particles was added to a 10 cm culture dish containing 2 × 10^6^ of HEK-292T cells. The DNA mixture was maintained in the cell culture for 6–8 h. Cell culture medium, i.e., Dulbecco Minimum Essential Medium (DMEM; Invitrogen, Carlsbad, CA, USA) was changed and cells were allowed to grow for 24 h at 37 °C in a humidified incubator in an atmosphere of 5–7% CO_2_. The medium was collected, filtered with 0.4 µm filter and supernatants were centrifuged at 3000× *g*, at 4 °C, for 10 min. Supernatant aliquots were frozen at −80 °C. One batch of DPCS and two of SHED cells were used for the transduction with lentivirus supernatant at early passages. A solution containing 2 mL of the supernatant, 6 mL of alpha-Minimum Essential Medium (α-MEM; Invitrogen, Carlsbad, CA, USA) and 4 µg/mL of polybrene (Sigma-Aldrich, St Louis, MO, USA) was added to the plated cells for 30 h. The efficiency of the transduction was checked using a standard fluorescent microscope followed by a confocal microscope (Leica inverted SP5—Leica microsystems; Wentzler, Germany) to check for the rainbow effect. The lineages created after the transduction were named by adding the word RGB after the initial name of the primary cell culture.

For color analysis, cells platted in coverslips were left for 30 min in a fixative solution containing 80 µL of 25% glutaraldehyde (Fisher Scientific; Waltham, MA, USA), 540 µL of 37% formaldehyde (Fisher Scientific, Waltham, MA, USA) and PBS (Invitrogen, Carlsbad, CA, USA) to top 10 mL. Coverslips were mounted on top of histological slides with Vectashield without DAPI (Vector Laboratories, San Francisco, CA, USA). Imaging was achieved using an inverted Leica SP5 confocal microscope (Leica microsystems, Wentzler, Germany). The 405 nm laser and the tunable white light laser were used for imaging cells which was sufficient to excite fluorescence in all three channels (red, blue and green). The detectors range were established as following: red channel spectral range was from 585–620 nm bandpass; green channel, a filter with 535–565 nm bandpass; blue channel, a filter with 465–500 nm. Each channel was acquired sequentially between lines. Images were recorded in 1024 × 1024 format and scanning speed was set to 100 Hz in bidirectional mode. The zoom factor varied according to the area analyzed, with the line average set to 4. Pinhole size was set to 50 µm.

### 2.2. Sprouting Formation and Morphology

DPSC-RGB I at passage 14 and 15, DPSC-RGBII at passage 10 and 11 and SHED nor-RGB at passage 8 and 9 and were used in all experiments, including independent repetitions. Sprouts were created by platting 1.5 × 10^4^ SHED-RGB or DPSC-RGBII into 350 μL/well (of 24 well plates) of growth factor reduced Matrigel (BD Biosciences; Franklyn, NJ, USA). For each experiment two wells per cell-line per time of analysis were seeded. Growth medium (α-MEM) supplemented with 15% fetal bovine serum (FBS, Invitrogen, Carlsbad, CA, USA) and 1% penicillin/streptomycin (Invitrogen) was used for the first 24 h. After that, it was changed into vasculogenic medium, i.e., Endothelial Growth Medium-2 for microvascular cells (EGM2-MV; Lonza) supplemented with 5% FBS (Invitrogen) and 1% penicillin/streptomycin (Invitrogen), as described [13]. Cells were allowed to grow and form sprouts for 7–11 days. As a control of the differentiation system, human dermal microvascular endothelial cells (HDMEC; PromoCell, Heidelberg, Germany) were used in the same experimental conditions. However, sprouts were already formed by 24 h in vitro with HDMEC. For the statistical analysis of sprouting numbers, five random areas/wells were marked on the plate using a previously created template. Photomicrographs of these areas were taken using microscope (Olympus; Tokyo, Japan) and Image Pro Plus software (version 5.1.2.59; Media Cybernetics Inc., Rockville, MD, USA) at 100×. The number of elongated capillary-like sprouts was counted per microscopic field. Two wells were used per time of analysis (7, 9 and 11 days) resulting in a total of 10 random fields counted per time-point and cell type. SPSS (IBM—Armonk, NY, USA) was used for statistical analysis. Data were presented as mean ± standard deviation. The t-test and two-way ANOVA, followed by the Bonferroni Test, were performed for comparing sprouting counts. *p* < 0.05 was considered statistically significant.

### 2.3. Imaging 3D Sprouts In Vitro

Custom-made chambers (2 mm thick) were created using a histological slide and a coverslip with a wax spacer to enable confocal imaging of sprouts (Supplemental Figure S1A). The coverslip size was used according to the need of the experiment and was sealed using warmed wax in three (out of four) sides leaving one open side for access (Supplemental Figure S1B). After transferring the matrigel containing sprouts to the chamber containing culture medium, the fourth side was also sealed with wax (Supplemental Figure S3C). Samples were analyzed using an inverted confocal microscope with coverslip turned down (Supplemental Figure S1D) within 48 h.

For imaging after 7–11 days of vasculogenic induction, sprouts generated by SHED-RGB cells were left for 2 h in a fixative solution containing 80 µL of 25% glutaraldehyde (Fisher Scientific), 540 µL of 37% formaldehyde (Fisher Scientific, Waltham, MA, USA) and PBS (Invitrogen, Carlsbad, CA, USA) to top 10 mL. Matrigel containing sprouts were transferred to custom slide chambers and one or two drops of Vectashield without DAPI (Vector Laboratories, Sao Francisco, CA, USA) was added before the chambers were sealed. Imaging for pictures was achieved using an inverted Leica SP5 confocal microscope (Leica microsystems, Wentzler, Germany). The microscope configuration was the same as described for the analysis of RGB populations. However, the pinhole size was set to 200 µm and Z-stack pictures were taken in selected areas.

For the statistical analysis of the color pattern pictures taken of sprouts fixed at 7–11 days were used. Thirty images were used for each experimental time. This number was established by power calculation (data not shown). The images were taken in the Leica SP5 inverted confocal microscope (Leica microsystems, Wentzler, Germany) with the same settings for all the images, only the focus was adjusted. Each image was subsequently opened in Fiji (public domain Java image processing program; NIH, Bethesda, MD, USA) and the colors were separated as red, green and blue greyscale pixel channels. The pixels per area of each channel was read separately and a number that designated the mean intensity of that specific channel was calculated by the software. The mean intensity is the grey scale value in that area divided by the total number of pixels. The areas of all images were the same, thus, the number of pixels of each channel was the only changeable variable. For the analysis of how colors changed over time (7, 9 and 11 days) univariate Anova followed by Tuckey test analysis was used. Pearson’s correlation tests were used to compare each color to the others, at the specific time points. SPSS (IBM—Armonk, NY, USA) was used for statistical analysis.

Imaging for movies created from Z-stack reconstructions of fixed sprouts at 7–11 days was performed using an inverted Nikon A1 confocal microscope (Nikon Corporation; Tokyo, Japan). The 405 nm, 488 diode and 561 diode lasers were sufficient to excite fluorescence in all three channels (Red, blue and green). Emission filters were fixed according to the microscope specification, as follows: red channel, a filter with 585–620 nm bandpass; green channel, a filter with 535–565 nm bandpass; blue channel, a filter with 465–500 nm bandpass. Each channel was acquired sequentially between lines. Images were recorded in 1024 × 1024-pixel format. The zoom factor varied according to the area analyzed, with the line average set to 4. Pinhole size was set to one airy unit.

Live cell tracking imaging was performed using SHED-RGB cells. Single areas and colors were obtained on time-lapse movies over a period of 2 days and 15 min of images taken every 1 h (49 cycles—from day 7.5 to day 9.5 after platting) and 43 sprouts were analyzed in total. Aligned time-lapse movies were used to manually identify cell gain or loss events, and cells were followed for movement in the sprout. Live imaging was performed using an upright Leica DMI6000 inverted fluorescence microscope (Leica Wentzler, Germany) fitted with Tokai Hit stage top incubator (INU series, D35-200F; Tokai Hit Co; Shizuoka-ken, Japan). An argon lamp fitted with filter cubes was used to excite fluorescence in all three channels (RGB). The filter cubes used were established as following: red channel (N3), a filter cube with 546/12 excitation and 565–620 nm bandpass; green channel (L5), 480/40 excitation and a filter with 505–565 nm bandpass; blue channel (I3), with excitation at 436/20 and a filter with 480/40 nm bandpass. Channels were acquired simultaneously.

### 2.4. In Vivo Transplantation and Treatment

All animal work was done under an UCUCA approved protocol using SHED-RGB cells. Male C57 SCID (Charles River; Boston, MA, USA) mice aged approximately 6 weeks old were used. The animals were divided into two groups. Group 1 (*n* = 6) was euthanized after 1 week of treatment and Group 2 (*n* = 6) after 2 weeks. Treatment started 2 weeks after cell’s transplantation plated into PLLA scaffolds and consisted of intra-peritoneal administration of 5 mg/kg Bevacizumab (Avastin; Genentech, San Francisco, CA, USA) every other day. Half of the animals were treated, and the other half were control and received only injections of saline solution. For transplantation, highly porous, poly-L-lactic acid (PLLA) scaffolds were prepared as described [23], cut into 6 × 6 × 1 mm squares and sterilized in 1 h cycles of ethanol 100% and 70%. For in vivo transplantation, 1 × 10^6^ cells resuspended in 10 µL of cell medium and 10 µL of Matrigel (BD Biosciences; Franklyn Lakes, NJ, USA) were seeded in each PLLA scaffold. After 30 min, scaffolds were transplanted into the subcutaneous space of the mice. When the experimental time was completed, the mice were euthanized, scaffolds containing cells were retrieved and fixed overnight at room temperature. For each group, two scaffolds were fixed for cryosection analysis and one for H&E staining. Two independent repetitions were done to verify reproducibility of the data. For cryosections, samples were mounted in OCT (Fisher Healthcare; Waltham, MA, USA), sectioned into 15 µm slides and analyzed by confocal microscopy as previously described. Samples were maintained protected from light all time.

## 3. Results

### 3.1. Pulpbow

**Figure 1:**
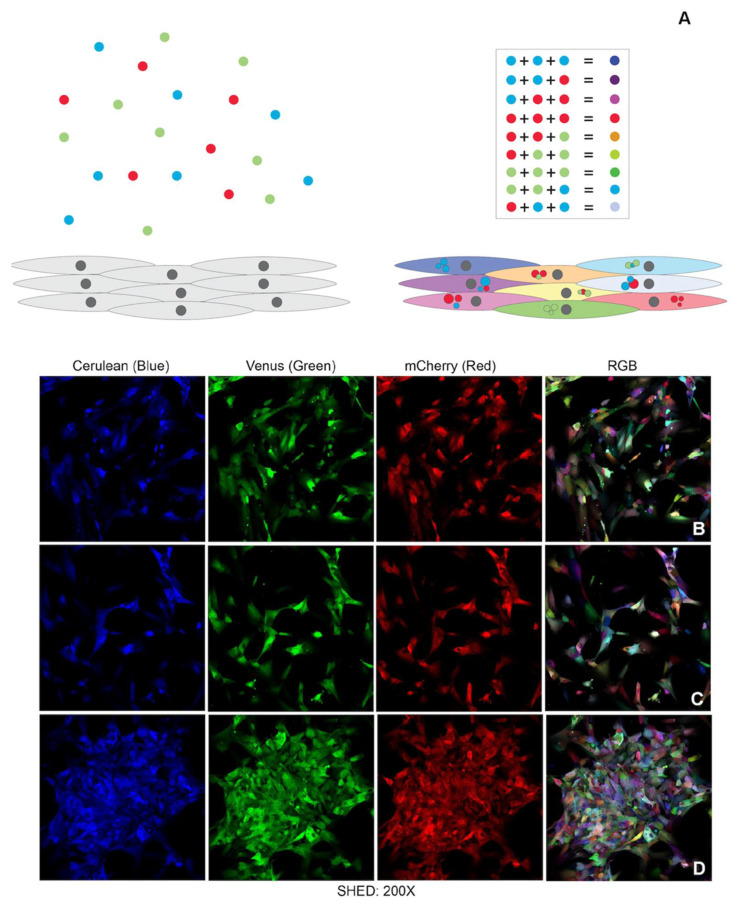
The pulpbow: (**A**) Schematic view on how RGB lentiviral vectors were incorporated in varying amounts inside single cells resulting in different combinations of red, blue and green fluorophores per cell creating the rainbow effect. (**B**–**D**) Separate channels show how these 3 colors are present in virtually all cells. The Pulpbow is formed by the merged channels (RGB) which allows the visualization of the rainbow effect.

The transduction of dental pulp stem cells with a random number of lentiviral copies of RNA encoding red (mCherry), blue (Cerulean) and green (Venus) generated the pulpbow for both DPSC and SHED cells (named DPSC-RGB I, DPSC-RGB II and SHED-RGB respectively), as depicted in Figure 1A–D. Confocal microscopic analysis immediately after transduction showed that both DPSC-RGB and SHED-RGB showed a vast array of colors (a rainbow) dispersed throughout the plates randomly (Figure 1B–D). When RGB channels were analyzed separately, virtually all cells expressed different amounts of red, blue or green (Figure 1B–D). Visual inspection of merged channels showed that no single color could be identified as more frequent than others. After cycles of freezing/thawing, cells did not show any sign of morphological or color alteration. Colors were stable, permanent and proliferating cells passed their color tags to their offspring. Vibrant colors could be found for at least 3 weeks after transduction (data not shown).

### 3.2. Pulpbow Sprouting

**Figure 2:**
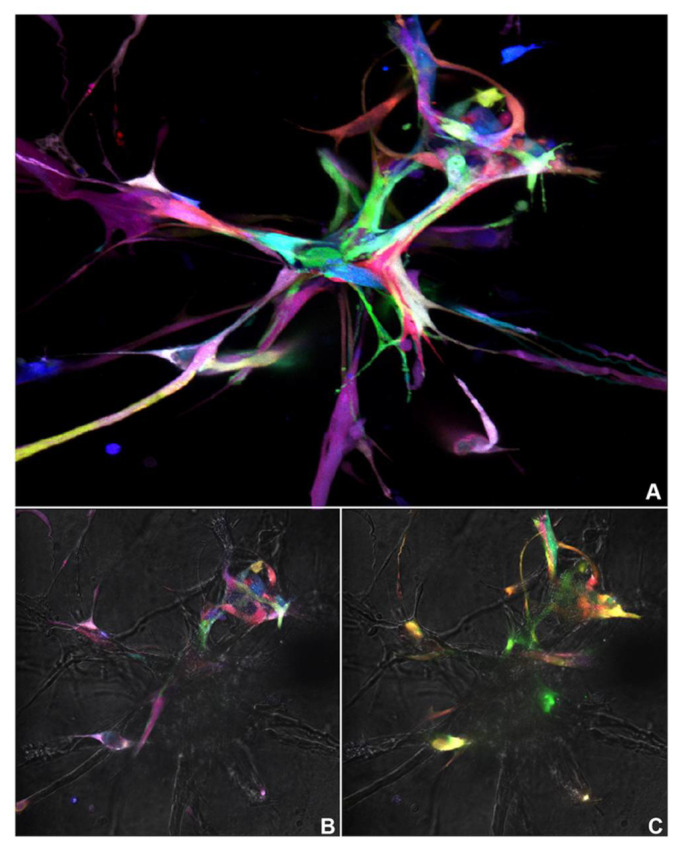
Color pattern analysis—Pulpbow Z-stack reconstruction of a 12-day old sprout from DPS—RGB I. (**A**) Snapshot of the Z-stack whole reconstruction (2D image). (**B**,**C**) Two Different areas of the same sprout during the acquisition of the Z-Stack.

To determine whether the Pulpbow could be used to study dental pulp stem cell-mediated vasculogenesis, DPSC-RGB I and II and SHED-RGB were induced to form sprouts in 3D gels. A Leica inverted SP5 confocal microscope was used to generate multiple images (2D analysis) that were used to generate Z-stack reconstructions in areas of robust sprouts (3D reconstruction). All cells induced by vasculogenic medium formed 3D capillary-like sprouts. As seen in the initial pulpbow analysis, sprouts were also formed by a vast array of colors randomly organized. Different areas of the sprouts were formed by different and multiple colors and cells touching each other had different colors (Figure 2A–C). DPSC-RGB I was originally from a more advanced passage compared to the other 2 RGB cell lines. In addition, visually it proliferated slower and formed less sprouts compared to DPSC-RGB II and SHED Nor RGB sprout formation. Thus, DPSC-RGB II and SHED nor-RGB were chosen for further experiments.

### 3.3. Sprouting Morphology over Time

**Figure 3:**
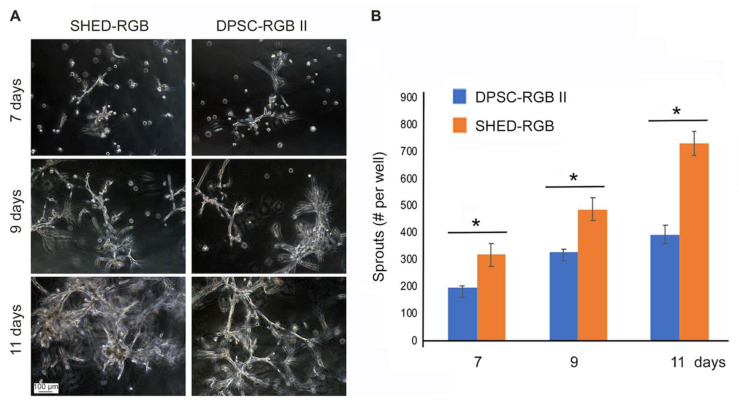
Morphological analysis: (**A**) sprouts formed by SHED-RGB and DPSC-RGB II at 7, 9 and 11 days. (**B**) Statistical analysis showed that SHED-RGB cells were more efficient in sprout formation than DPSC-RGB II. Magnification bar depicted is the same for all images in panel A. Asterisks depict statistical significance at *p* < 0.05.

SHED-RGB and DPSC-RGB II were induced to endothelial differentiation through formation of sprouts in three dimensions (3D) inside Matrigel (Supplemental Figure S1). Sprouts were fixed after 7, 9 and 11 days for both morphological and color pattern analysis. At 7 days of induction with vasculogenic medium, cells of both lineages started to form clusters that varied in sizes. Not all cells plated were involved in the clusters and many single cells could be seen spread throughout the plates. At 9 days, cells started to emerge from the clusters and dive into the Matrigel forming areas of elongations and at 11 days, these areas were longer, thicker and many times touching each other (Figure 3A). Even though both cell lines responded similarly in the morphological analysis, it was clear that DPSC-RGB II started sprouting earlier than SHED-RGB. But, once elongations were formed, they soon became more abundant and longer in SHED nor RGB cells than in DPSC-RGB II. Statistical analysis (Figure 3B) using unpaired *t*-test showed that the difference in sprout numbers comparing DPSC-RGB II and SHED-RGB was statistically significant in all days analyzed (*p* = 0.0056 at 7 days, *p* = 0.0034 at 9 days and *p* = 0.0046 at 11 days). When these two cell types are compared over time by ANOVA followed by Bonferroni test, only at day 11 the SHED-RGB growth was statistically significantly higher than DPSC-RGB II (*p* < 0.0001). Thus, SHED-RGB cells were selected for further analysis.

### 3.4. Color Pattern Analysis over Time

**Figure 4:**
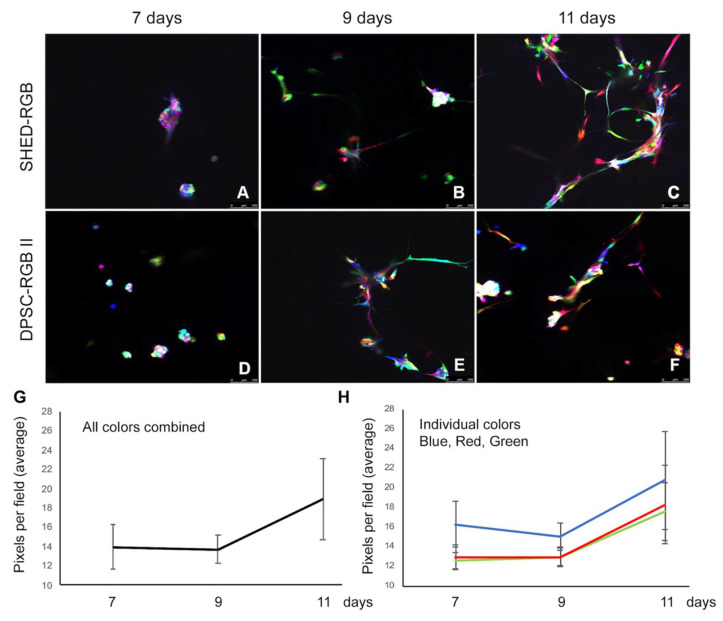
Color pattern analysis over time—Both cell lines showed a vast array of random colors in sprouts fixed at 7 days (**A**,**D**), 9 days (**B**,**E**) and 11 days (**C**,**F**). It is possible to see single cells not involved in sprout formation at 7 and 9 days but not at 11 days. (**G**) Statistical analysis showed that at 11 days there was significant more areas of the plates populated by colored cells (*p* = 0.000) while 7 and 9 days, there was no statistical difference. (**H**) Blue was more predominant than red and green when the colors were analyzed separately and over time, the difference between pixels per area of these colors were statistically significant at 11 days (*p* < 0.0001). There was no significant difference between red and green.

The color pattern was further analyzed over time using confocal microscopy in sprouts derived of SHED-RGB cells fixed at 7, 9 and 11 days. A Leica inverted SP5 confocal microscope was used to generate photomicrographs (2D analysis) and Z-stack reconstructions of areas of robust sprout formation (3D reconstruction) while a Nikon A1 inverted confocal microscope was used to make 3D movies (also obtained from z-stacks) but using tools to rotate and walk up and down the sprout areas. Three independent repetitions were performed for all color analysis experiments. Visual inspection of capillary-like sprouts analyzed for both cell types showed that there was no single cell progeny (identified by color) that was more prone to differentiate into vascular endothelial cells. A vast and random array of colors could be found both in the initial cell clusters and in elongation areas (Figure 4A–F). Interestingly, in the plates of sprouts fixed at 7 and 9 days, not all cells were forming capillary-like sprouts. At early stages, cells were organized as clumps or elongated single cell dispersed throughout the plates. However, at 11 days, most cells were organized into capillary-like sprouts and rare single cells could be observed in the 3D gels.

To further investigate the color pattern, we proceeded with statistical analyses. Colors were translated into numbers using the software Fiji (NIH, Bethesda, MD, USA) to separate color channels and read the pixels per area of red, green and blue wavelengths. One-way ANOVA followed by the Tukey Test was used to compare average pixels per area of red, blue and green, to investigate how average of all colors (all channels together) and how separate channels of red, blue and green changed over time. These results are depicted in Figure 4G,H respectively. On average, there are more pixels per area of all colors at 11 days when compared to 7 and 9 days (*p* < 0.0001—Figure 4G). The aggregated blue color (pixels per area of blue wavelength) was more predominant than the green and red ones (*p* = 0.000); and is also more prevalent at 11 days when there are more pixels per area of all colors (*p* = 0.000, Figure 4H). Pearson’s correlation test was used to compare two sets of color over time and showed that there is statistically significant correlation among all colors and at all times analyzed (Supplementary Figure S3).

### 3.5. Organization by Proximity

**Figure 5:**
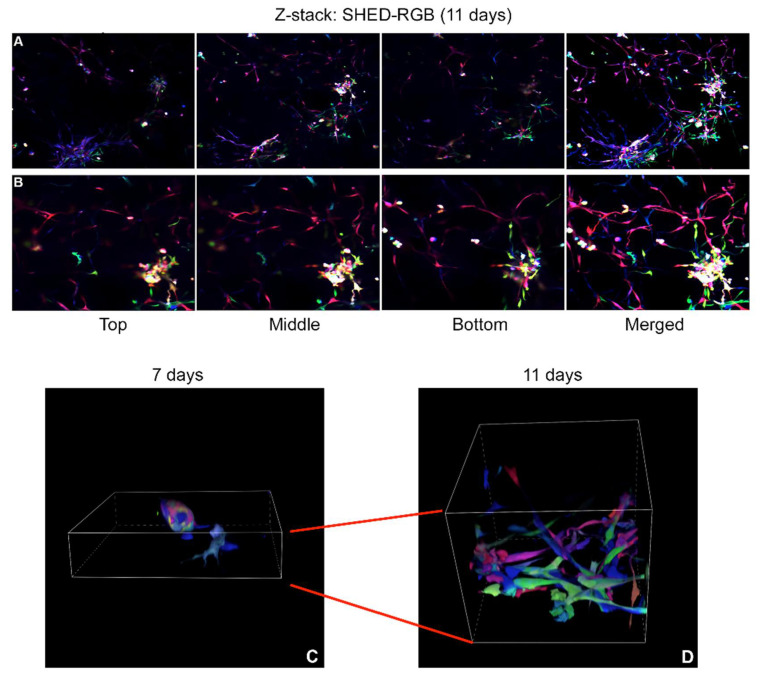
Organization by proximity—3D Z-Stack reconstruction showing groups same-colored cells organized by proximity in different areas of the sprout. All images in panel A refers to one sprout area formed by different clusters of cells with elongations intercommunicating them. All images in panel B refers to a detail of this sprout area. From left to right it is possible to see the top, middle and bottom z-stack images of these sprouts and the very right image is the merged snapshot of the whole z-stack reconstruction. (**A**) Top—it is possible to see three different clusters of same colored cells in different areas of the sprout: bluish cells in the lower left area, green cells in the middle right area and red cells in the upper left area; Middle—it is possible to see elongations formed by red cells on the mid-top of the image and on the lower left, elongations formed by pink and purple cells and a cluster of yellowish cells in the upper right corner; Bottom—it is possible to see a group of green cells in the lower right corner and some elongations formed by red cells. (**B**)—Top and Middle—Detail of red cells forming elongations in the mid-top areas and the cluster of yellowish cells in the lower let areas; Bottom—a group of green cells appearing under the cluster of yellow cells. Merged images of these sprouts clearly show clusters and elongations formed by same-colored cells touching each other’s located in specific areas of the sprouts. Supplementary Videos S1 and S2 (Movies 1 and 2) depicts the z-stack 3D reconstruction of these two panels and contain the magnification bar. (**B**) is a detail of (**A**). (**C**,**D**) Snapshots of the 3D reconstructions obtained from movies of sprout areas induced for 7 (**C**), and 11 (**D**) days. At 7 days (**C**) small groups of cells and rare elongations can be seen while at 11 days (**D**), sprouts increased in heigh, width and depth and are formed numerous elongations. Supplementary Videos S3 and S4 (movies 3 and 4); Supplementary Videos S5 and S6 (movies 5 and 6) show these 3D reconstructions both as build-up and rotations areas respectively.

SHED-RGB cells were used for further analyses as they were more efficient in capillary-like sprout formation. When Z-Stack 3D reconstructions of more robust sprouts (differentiated for 11 days) were further studied, a co-localization pattern could be observed as groups of same color cells were organized by proximity, suggesting that progeny cells organize themselves into multi-cellular capillary-like sprouts (Figure 5—panels A and B, and Supplementary Videos S1 and S2 (movies 1 and 2). This organization by proximity was confirmed in movies from 3D reconstructions of sprout areas for all experimental times (7, 9 and 11 days). A Nikon A1 inverted confocal microscope was used with rotation and addition/subtraction tools that facilitate the visualization of different focal planes inside the sprouts. Supplementary Videos S3 and S4 (movies 3 and 4) show sprouts fixed at 7 days; Supplementary Videos S5 and S6 (movies 5 and 6); sprout fixed at 9 days and Supplementary Videos S7 and S8 (movies 7 and 8) sprouts fixed at 11 days. These movies clearly show the 3D growth of the sprouts over time as the area of the Matrigel occupied by each sprout was progressively bigger and thicker (Figure 5C,D).

### 3.6. Color Pattern Analysis of Live Cells

To further investigate how the SHED-RGB cells interact with each other while forming sprouts, we performed a time lapse study using live cell sprouts from day 7.5 to 9.5 capturing images every hour for 48 h. Our results showed once more that there was a vast array of colors participating in the sprout formation. It was very clear that there was intense and fast movement in all 43 movies analyzed, by the end of the experiment, most cells had moved out of the area of analysis set as the focal point of the microscope. Some sprouts (cells) can be seen for a longer time than others as cells moved away at different paces. In addition, during the experimental time, some colors fade away before others leaving a blurry bluish/purple image of the cell’s structure at the end of the experimental. These characteristics make the analysis of these experiments complex. However, when specific areas of robust sprouts were analyzed, a color pattern specially in the elongations could be found as same-colored cells were close to each other and even touching each other. These results can be seen in Supplementary Video S7 (movie 7—fast moving cells and clumps) and Supplementary Video S8 (movie 8—moving cells and elongations with green predominant).

### 3.7. In Vivo Analysis

**Figure 6:**
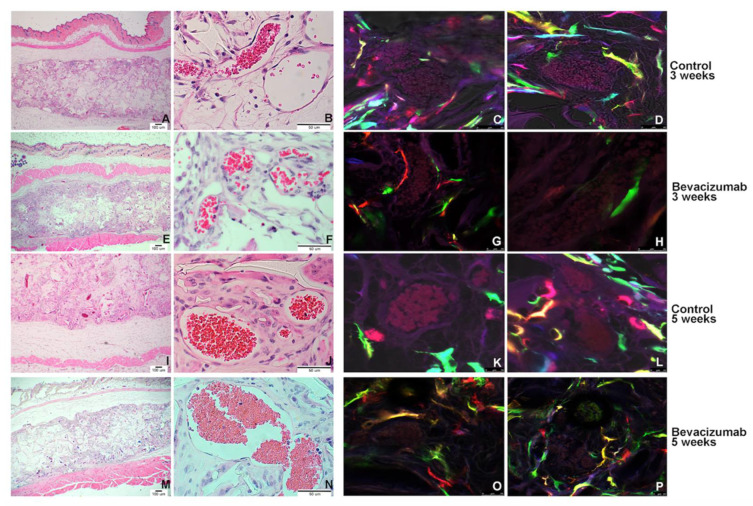
In vivo transplantation—Morphological analysis of transplanted SHED Nor cells (**A**,**B**,**E**,**F**,**I**,**J**,**M**,**N**). HE stained sections showing control (**A**,**B**,**E**,**F**) and treated (Bevacizumab injected) samples (**I**,**J**,**M**,**N**). In all groups it was possible to easily identify the transplanted areas in all samples and blood vessels were also evident. The blood vessels of samples collected after 3 weeks (Group 1—(**A**,**B**,**E**,**F**)) had thinner walls, and hemorrhage was more evident when compared to samples collected after 5 weeks (Group 2—(**I**,**J**,**M**,**N**)) that had thicker walls and not a lot of RBCs dispersed throughout the connective tissue. When the color pattern of the samples was analyzed by confocal microscopy, a vast number of colored cells were found independently of the treatment or time of analysis. Colored cells were also found surround blood vessel both in the first or second layer of cells in all samples (**C**,**D**,**G**,**H**,**K**,**L**,**O**,**P**). Supplemental Figure S2 depicts the technique used for in vivo transplantation, the macroscopic appearance at the collection day and the general aspect of the transplanted cells in the scaffold using only the green channel.

SHED-RGB cells were transplanted into immunodeficient (SCID) mice to validate if the pulpbow was suitable for in vivo transplantation and for studies aiming to understand if and how the transplanted RGB cells would contribute to dental pulp stem cell-mediated vasculogenesis. Poly-lactic acid scaffolds (PLLA) containing cells were observed for 3 and 4 weeks after the transplantation and 1 and 2 weeks after initiation of treatment with Bevacizumab (VEGF inhibitor) respectively. Two independent repetitions were analyzed. The morphological analysis performed in the histological H&E-stained sections showed that all samples (control and experimental groups) presented well-formed blood vessels (Figure 6A,B,E,F,I,J,M,N). It was possible to clearly identify the transplanted areas due to the presence of the scaffold (Figure 6A,E,I,M). At the most internal areas of the scaffolds containing SHED-RGB cells, it was possible to recognize a loose connective tissue, small and more sporadic blood vessels, remnants of the scaffold and some hemorrhage. At its most external areas, the connective tissue was denser, blood vessels of greater dimensions (diameter) and a conspicuous presence of multinucleated giant cells was noticed in some samples (Figure 6A,E,I,M). The loose connective tissue area decreased while the area of dense connective increase in samples collected from 3 to 5 weeks after transplantation respectively (Figure 6B,F compared to Figure 6J,N). The color analysis by confocal microscopy showed that blood vessels formed in the transplantation areas were a mix of vessels derived from the host (mouse) cells and vessels derived from the SHED-RGB cells that were transplanted. These cells could be found in the first or second layer of the blood vessel walls in all times analyzed and in both, control and experimental groups. The RGB cells surrounding blood vessels were of multiple colors and random sizes and morphology (Figure 6C,D,G,H,K,L,O,P). We did not observe major differences in the presence (or absence) of SHED-RGB cells or in the pattern of colors when we compared the control (Figure 6C,D,K,L) with the experimental (Bevacizumab) groups (Figure 6G,H,O,P) at 3 weeks or 5 weeks after transplantation.

## 4. Discussion

We developed a new effective tool to study the biology of dental pulp stem cells that allowed us to follow multiple individual or groups of cells over time: The Pulpbow. In addition, we used pulpbow populations derived from permanent (DPSC-RGB II) and deciduous teeth (SHED-RGB) to improve our understanding of mechanisms involved in vasculogenic differentiation of dental pulp stem cells in 3 dimensions (3D) over time. These populations were used in multiple experimental conditions and an assorted combination of methods, but always resulted in capillary-like sprouts formed by a wide and relatively random array of colored tagged cells. No experimental condition or method resulted in a single-colored sprout formation. Thus, it is reasonable to suppose that sprout formation mediated by dental pulp stem cells is a multicellular and likely a polyclonal process.

In attempt to further investigate if there was a pattern of color organization, we performed a study in which we focused on the analysis of the blue, red or green color that surprisingly showed blue as a predominant hue. Color perception is complex but blue is usually harder to see than colors of longer wavelengths such as red and green [24] and blue sensing cones comprise only 10% of the color sensitive cells [25]. This may explain why visually we could not perceive the higher frequency of blue cells when analyzing our results. In addition, even though blue was more prevalent, it was only one part of the final color that a cell was showing up, making it difficult for our eyes to distinguish only this wavelength. Pearson’s correlation analysis showed that there was statistically significant correlation comparing any two sets of color in any time point of the analysis (7, 9 or 11 days). This may be the result of high transduction efficiency of the lentiviral vectors leading to virtually all cells formed by all three colors.

Capillary sprout assays over time (7, 9 and 11 days) showed that not all cells formed sprouts, demonstrating that at least in the initial phases of dental pulp stem cell-mediated vasculogenesis, some cells or a population of cells is more genotypically and/or phenotypically primed for endothelial cell differentiation. At 7 days, we could find several single cells not involved in sprout formation. These cells were rarer at 9 days and nearly absent at 11 days. Further investigation is necessary to understand the characteristics of the cells that respond earlier compared to the ones that respond later. However, morphologically, it seems that with the persistency of vasculogenic stimuli, many individual dental pulp stem cells are capable to respond to the vasculogenic differentiating stimuli.

Our results also showed that sprout formation mediated by dental pulp stem cells is a continuous process that started with formation clusters of cells (mostly seen at 7 days) that subsequentially formed elongations from the cell clusters (seen at 9 and 11 days). The clusters and elongations eventually increased in size and numbers and showed multiple layers of cells eventually resulting in inter-communicating sprouts (mostly seen at 11 days). As stated, this process took multiple days from cell’s plating day 0 to the first signs of differentiation. In contrast, when we used primary human endothelial cells (HDMEC) as controls, it took less than 24 h to form capillary-like sprouts. We hypothesize that the reason for this delay is that dental pulp stem cells needed to be induced to first differentiate into vasculogenic endothelial cells to form the capillary-like sprouts, while HDMEC cells are already differentiated endothelial cells.

The need for differentiation before function show that these cells are forming sprouts through vasculogenesis (not angiogenesis). In this regard, our data corroborates with Teper and colleagues (2005) that used an ischemic model of angiogenesis to recruit circulating bone marrow endothelial progenitors from the blood to ischemic areas. Cells initially proliferated in clusters. By day 14, these clusters coalesced into vascular cords and became functional vessels by day 21. According to the authors, the sequence of recruitment, proliferation and differentiation of mesenchymal stem cells corresponds to vasculogenesis, is markedly different from the events that characterize angiogenesis, and is similar to the events that happen during inflammatory response [26].

Movies obtained from three-dimensional (3D) z-stack reconstructions of SHED-RGB derived sprouts at 7, 9 and 11 days after the initial induction showed that different cell colors could be found in similar focal planes of the Matrigel pointing out to a pattern of organization by proximity. This result was confirmed by the 3D reconstructions obtained from robust sprout areas fixed at 11 days that showed the organization by proximity but also showed multiple groups of same color cells located in small areas, indicating that the initial organization is followed by a clonal expansion. Time lapse movies following SHED-RGB cells for 2 days (from 7.5 to 9.5 day) showed intense movement of cells in different directions. Even though the movies followed cells for 2 days, in several movies just after a few hours, there were no cells left in the focal plane as they moved away from it. Interestingly, as seen in the movies obtained from 3D (Z-stack) reconstructions from sprouts fixed at 7, 9 and 11 days, the time-lapse movies also showed areas of same-colored cells touching each other or in the same focal plane.

The Pulpbow cells that initially responded by intense migration towards the sprouts seen in our results, might be cells primed to vascular differentiation such as the VEGFR1-positive population described by Bergamo and colleagues [17]. We hypothesize that once the primed cells undergo vasculogenic differentiation and others might be necessary to continue the blood vessel formation, local clonal proliferation such as seen in our study may respond to the cell demand. Further research is necessary to confirm this hypothesis.

Dental pulp stem cells have been previously associated with pericytes but the extent that pericytes act as MScs has long been a contentious issue. Previous in vitro and in vivo studies have shown that dental pulp stem cells often express protein markers shared by pericytes [1,2]. This makes sense from an evolutionary perspective since the location of stem cells in proximity to blood vessels can facilitate migration and response to injury. However, when genetic labeling and lineage tracing strategies were used to investigate pericytes as MSCs, a dual origin (one pericyte and other non-pericyte) was found in dental tissues [27]. Endothelial cells and pericytes are closely associated. DPSCs treated with TGF-β1 can differentiate into functional pericyte-like cells after induction and vessels formed by these cells maintain stability through Ang1/Tie2 and VEGF/VEGFR2 signaling [28]. Culture conditions can also modulate expression of pericyte markers in DPSCs as well as influence their capacity to stabilize sprouts. DPSC cells cultured in standard conditions expressed MSC markers (CD44, CD90, CD105 and CD73) and contained a population of NG2 positive pericytes with low sprouting capacity. When the same cells were cultured in vasculogenic medium, the NG2 population was up-regulated and the sprouting capacity increased [29]. It is important to note that in our in vitro experiments, cells were cultured in vasculogenic medium and thus, we predict that our sprout-forming population was enriched with pericytes.

Finally, we used our pulpbow system (SHED-RGB) for in vivo transplantation. Even though our focus was blood vessels, we could see again a wide and random array of colored cells occupying the whole transplanted scaffold. Color-tagged cells could also be found surrounding blood vessels as the first layer of cells assuming an endothelial-like morphology or the second layer of cells with pericyte appearance. In both cases, different cell colors were found even when a VEGF inhibitor (Bevacizumab) was used to block the VEGF signaling pathway. This result once more point out to a “plasticity” capacity as several types of cells could be induced to form blood vessels even in impaired conditions.

Due to the easily accessible nature and high proliferative rates, dental pulp stem cells are excellent sources of multipotent MSCs both for research purposes and future uses in tissue engineering. Mesenchymal stem cells from different origins show similar basic characteristics and behaviors when induced in vitro even though their origins and functions in vivo may be different. Moreover, the circulatory system is unique in the body meaning that blood vessels formed in a tooth or other parts of the body follow the same mechanisms. Consequently, we expect that our results may likely be true in other human tissues and mesenchymal stem cell populations.

As with any new technology, there are limitations to the labeling process since it cannot be fully controlled. Even when cell numbers are controlled before viral transduction, we cannot control the number of viral particles that will enter each cell and the integration sites inside the cell’s DNA. Thus, after transduction with the LeGO vectors, we don’t know the initial number of cells of particular color and if the initial population was formed by equal proportions of hues (colors). The lack of control of how the RGB viruses integrate into the cells may also result in the same progenitors being marked with different colors. In this case, different colored cells would have the same origin and multicolored sprouts could still be monoclonal. Furthermore, when passing the RGB cells, we cannot predict if we are selecting a determined color over the others. To try to minimize all these limitations, before starting this research project, cells were expanded immediately after the transduction to create the same pool of cells for all experiments.

Collectively, dental pulp stem cell-derived vasculogenesis is completed by cells showing a vast array of colors (multicellular process, possibly polyclonal), that start with an initial period of differentiation that takes around 5–7 days until an initial morphological structure (clusters of cells) can be seen. This period is followed by the formation of cell elongations in a phase characterized by intense cell mobility to and from the cell clusters. Further movement is noticed until more robust sprout structures are formed, and individual cells start to proliferate locally showing same-colored cells organized by proximity (local clonal expansion).

## Supplementary Materials

The following are available online at https://www.mdpi.com/article/10.3390/cells10112804/s1, Supplementary Figure S1—Technique used to fabricate the custom-made slide chambers. Supplementary Figure S2—In vivo transplantation technique. Supplementary Figure S3—Pearson’s correlation results. Supplementary Video S1—Movie 1. Supplementary Video S2—Movie 2. Supplementary Video S3—Movie 3. Supplementary Video S4—Movie 4. Supplementary Video S5—Movie 5. Supplementary Video S6—Movie 6. Supplementary Video S7—Movie 7. Supplementary Video S8—Movie 8.
