# Supplementary material for: Pulpbow: A Method to Study the Vasculogenic Potential of Mesenchymal Stem Cells from the Dental Pulp
Source: Cells. 2021 Oct 20;10(11):2804. doi: 10.3390/cells10112804 (PMC8616523; doi:10.3390/cells10112804)
Supplement: Supplementary file 1 [file cells-10-02804-s001.zip › Supplemmentary information-Mantesso et al.pdf]

Supplementary information – Mantesso et al

Pulpbow: A Method to Study the Vasculogenic Potential of Mesenchymal Stem Cells from the Dental Pulp

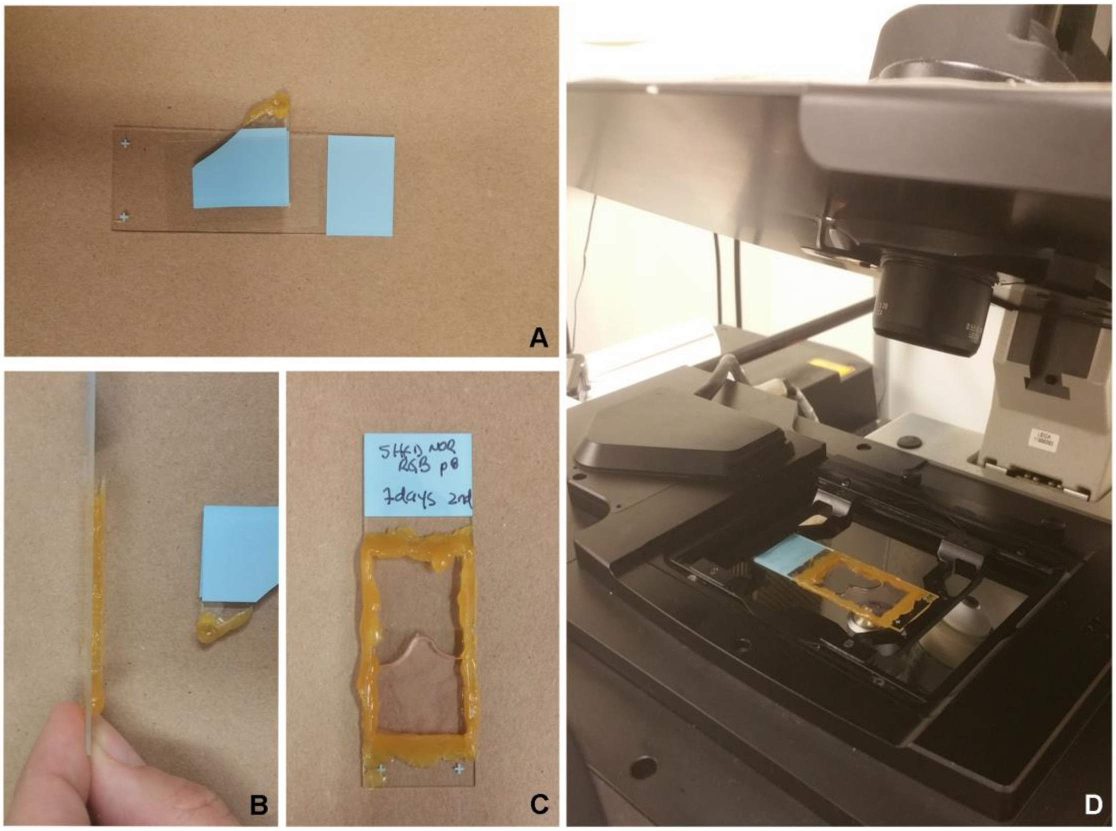

Supplementary Figure S1 – Representation of the method used to fabricate the custom-made slide chambers. A and B - Preparation technique. C – Matrigel containing cells grown in 3D. D – Use in the microscope with the coverslip turned down.

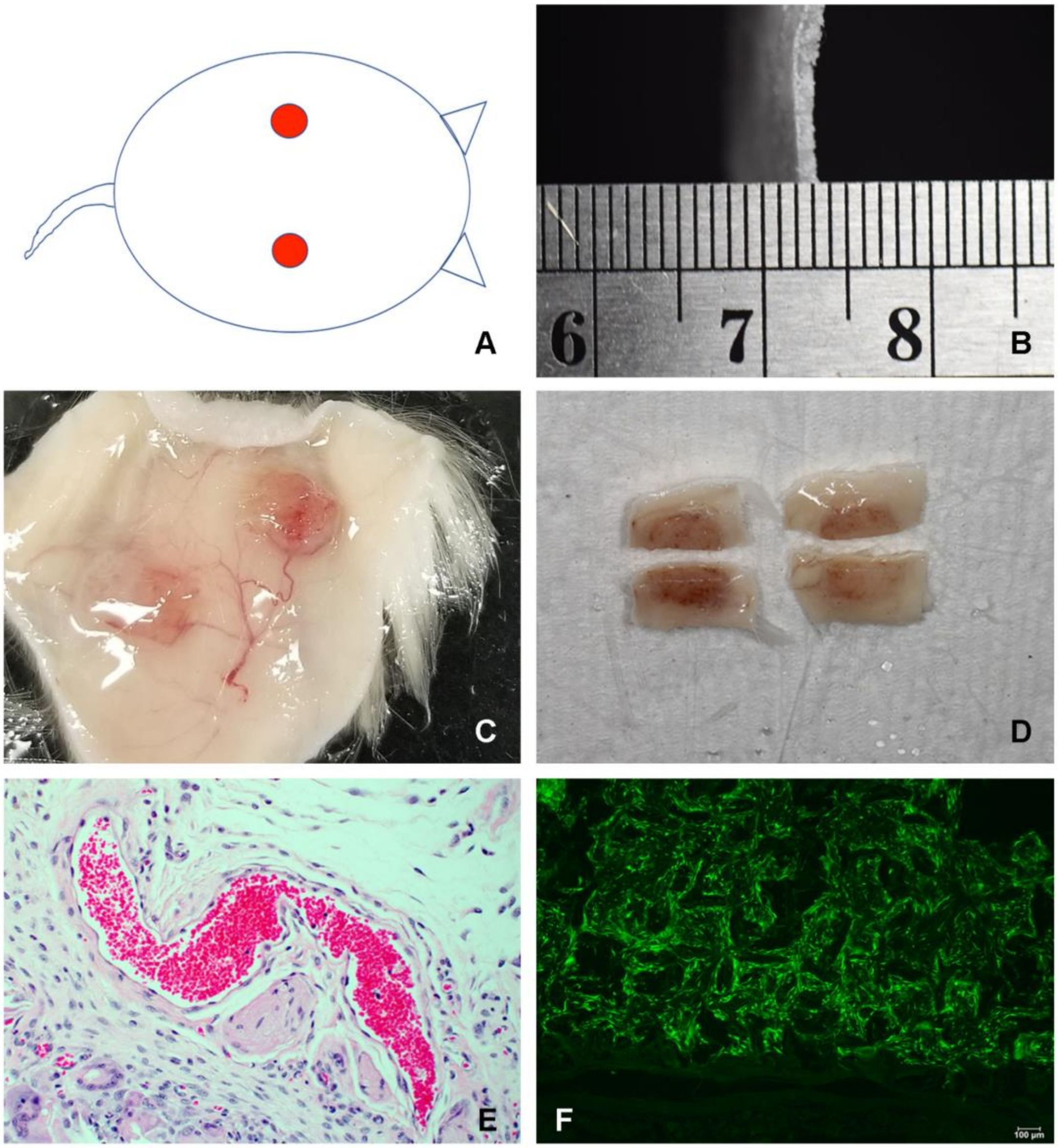

Supplementary Figure S2 – *In vivo* transplantation technique and fluorescent microscopic view of the transplantation area. A – Schematic representation of the areas of transplantation. Each mouse received 2 scaffolds. B – PLLA scaffold aspect and measurement before use. C-D – Macroscopic appearance of the transplants after 5 weeks *in vivo*. Before fixation (C) and after fixations (D). E – Histological aspect of the main neurovascular bundle that nourishes the transplanted area (marked with * in C). F – Simple fluorescence (green channel only) showing the amount of transplanted SHED Nor RGB cells in one scaffold.

A

Correlations[a]

		MeanR	MeanG	MeanB
MeanR	Pearson Correlation	1	.914**	.946**
	Sig. (2-tailed)		.000	.000
	N	30	30	30
MeanG	Pearson Correlation	.914**	1	.838**
	Sig. (2-tailed)	.000		.000
	N	30	30	30
MeanB	Pearson Correlation	.946**	.838**	1
	Sig. (2-tailed)	.000	.000	
	N	30	30	30

**. Correlation is significant at the 0.01 level (2-tailed).

a. Day = 7

Correlations[a]

		MeanR	MeanG	MeanB
MeanR	Pearson Correlation	1	.593**	.801**
	Sig. (2-tailed)		.001	.000
	N	30	30	30
MeanG	Pearson Correlation	.593**	1	.786**
	Sig. (2-tailed)	.001		.000
	N	30	30	30
MeanB	Pearson Correlation	.801**	.786**	1
	Sig. (2-tailed)	.000	.000	
	N	30	30	30

**. Correlation is significant at the 0.01 level (2-tailed).

a. Day = 9

Correlations[a]

		MeanR	MeanG	MeanB
MeanR	Pearson Correlation	1	.886**	.840**
	Sig. (2-tailed)		.000	.000
	N	30	30	30
MeanG	Pearson Correlation	.886**	1	.923**
	Sig. (2-tailed)	.000		.000
	N	30	30	30
MeanB	Pearson Correlation	.840**	.923**	1
	Sig. (2-tailed)	.000	.000	
	N	30	30	30

**. Correlation is significant at the 0.01 level (2-tailed).

a. Day = 11

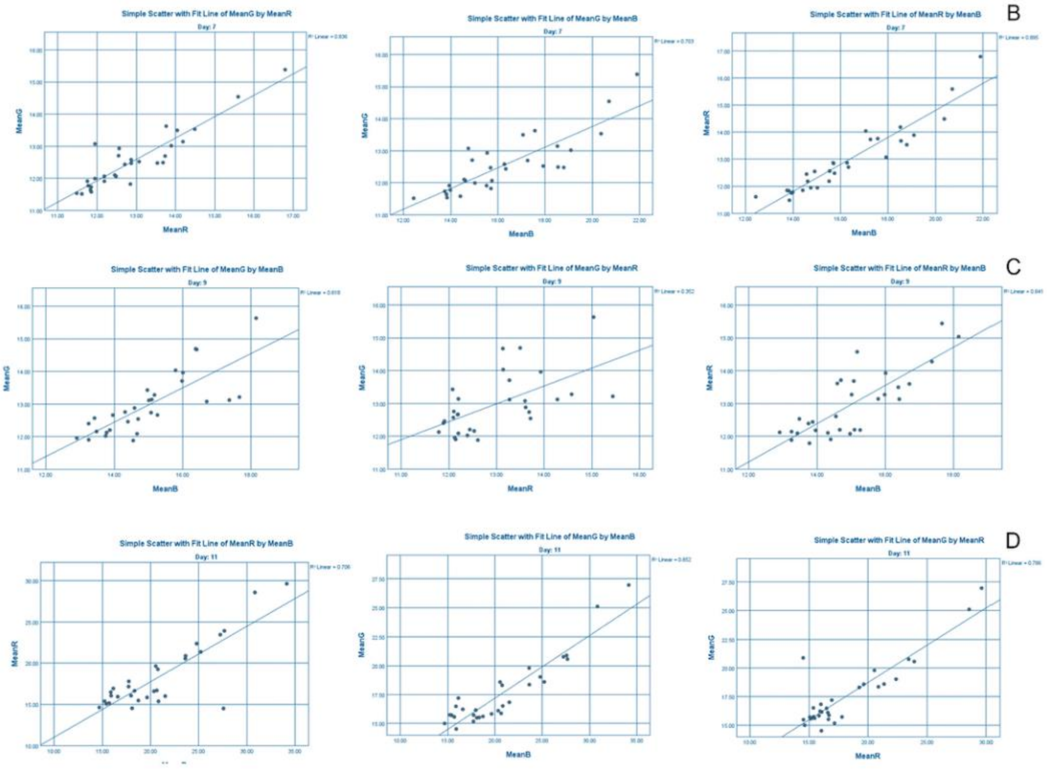

Supplementary Figure S3 - Pearson's correlation tests by day showed that there is statistically significant correlation among all colors and at all times analyzed. A – Table depicting the mean values of the pixels per area of blue wavelength of Red (R), Green (G) and Blue (B) light waves. The correlation coefficient of the mean values for each color is one or close to one. B – D – Scatter plots for 7 day comparisons (B), 9 day comparisons (C) and 11 day comparisons (D) demonstrating that the average value of a color accompanies the average value of the other colors and that most readings are close to the correlation line specially for 7 (B) and 11 (D) days readings.

Legends-Movies

Supplementary Video S1 (**Movie 1) –** 3D reconstruction of a robust sprout fixed 11 days after the beginning of vasculogenic differentiation. As the focus move from top to bottom of the 3D sprout, groups pf same-colored cells appear in groups at specific locations. The group of the green cells (middle right) that is on the focus on the top of the z-stack is progressively clearer then disappears while a group of yellow cells and groups of red (upper half) and bluish elongations (lower left) start to be clearer on the focus plane. Almost at the end of the 3D reconstruction, a group of green cells is seen together again.

Supplementary Video S2 (**Movie 2) –** Detail of the upper right corner of the same sprout**.** As the focus move from top to bottom of the 3D sprout, groups of green (lower right) start to disappear while, a group of yellow cells located under the green cells can be seen. Towards the end of the reconstruction, it is possible to see a group of green cells showing up of the focus plane. Red cells forming elongations and a small group of bluish cells also forming elongations can be seen throughout the 3D reconstruction.

Supplementary Video S3 (**Movie 3) –** Three-dimensional build-up reconstruction of sprouts induced for 7 days. Mainly single cells or small groups comprised of a few cells can be seen different colors. Sprout elongations are rarely seen. Many different colors can be seen inside the 3D reconstruction area.

Supplementary Video S4 (**Movie 4) –** Same sprout induced for 7 days seen in a rotation view.

Supplementary Video S5 (**Movie 5) –** Three-dimensional build-up reconstruction of sprouts induced for 11 days. Elongations and cells of a wide range of colors can be seen. Groups of same-colored purple, blue, pink and green cells can be seen touching each other.

Supplementary Video S6 (**Movie 6) –** Same sprout induced for 11 days seen in a rotation view confirming the close contact among same-colored cells.

Supplementary Video S7 (**Movie 7) –** Live cell imaging of SHED-RGB from day 7.5 to 9.5 after induction. Cells move fast and in different directions trying to form connections.

Supplementary Video S8 (**Movie 8) –** Live cell imaging of SHED-RGB from day 7.5 to 9.5 after induction. Cells move fast and in different directions trying to form connections and it is also possible to notice that most cells forming elongations are green.
